# Digesting the Impact of Diet on Irritable Bowel Syndrome (IBS): Exploring Solutions for Controlling IBS

**DOI:** 10.7759/cureus.45279

**Published:** 2023-09-15

**Authors:** Sai Suseel Sarvepalli, Shree Laya Vemula, Saikumar Aramadaka, Raam Mannam, Rajagopal Sankara Narayanan, Arpit Bansal, Vishnu R Yanamaladoddi

**Affiliations:** 1 Department of Research, Narayana Medical College, Nellore, IND; 2 Department of Research, Anam Chenchu Subba Reddy (ACSR) Government Medical College, Nellore, IND; 3 Department of Internal Medicine, Narayana Medical College, Nellore, IND; 4 Department of General Surgery, Narayana Medical College, Nellore, IND; 5 Department of General Surgery, Narayana Medical College and Hospital, Nellore, IND

**Keywords:** constipation-predominant ibs (ibs-c), diarrhea-predominant ibs (ibs-d), fodmap diet, ibs treatment, treatment of ibs, ibs

## Abstract

Irritable bowel syndrome (IBS) plagues nearly a fifth of the general population. It is a chronic illness that can significantly lower quality of life (QoL) and work productivity. The relationship between diet and the functional gastrointestinal (GI) symptoms present in IBS is gaining more and more attention. In addition to being a factor in the pathophysiology of IBS, diet also has a significant impact on symptoms and overall well-being. Recent research has also shown that short-chain fermentable carbohydrates increase colonic gas production and small intestine water volume, which in turn causes functional GI symptoms in those with visceral hypersensitivity. This review article has consolidated various studies highlighting the association between certain foods and the pathophysiology of IBS. It has also talked about how restricting certain food items from the diet of affected individuals can relieve symptoms and in some cases can be more effective than pharmacotherapy. Although the low reduced fermentable oligosaccharide, disaccharide, monosaccharide, and polyol (FODMAP) diet (LFD) is a well-known method of treating IBS symptoms, over a third of individuals do not benefit from it. This article has also discussed the effectiveness and applicability of the LFD compared to other dietary therapies for the long-term management of IBS.

## Introduction and background

Irritable bowel syndrome (IBS) is a chronic functional gastrointestinal disorder (FGID) and is a very common diagnosis in clinical practice. The term "mucous colitis" was coined by Osler in 1892 [1]. He described a condition marked by the passage of tubular casts of the colon made up of mucus (mucorrhea), cell debris, and "intestinal sand." Osler said that the colonic epithelium was healthy and that many of the patients had colicky gastrointestinal symptoms and were hysterical, hypochondriac, or depressed. Hurst also identified this ailment, but by the late 1920s, both clinical practice and medical texts seemed to have forgotten about the illness they had described. By 1928, colonic spasm was the only condition that the name "mucous colitis" described. In 1929, Jordan and Kiefer coined the phrase "irritable colon" to refer to a colonic musculo-nervous disturbance that 30% of gastroenterology outpatients experience. The phrase "irritable colon" originally meant "abdominal pain and disordered defecation," and its current meaning is thus comparable to it [2].

Despite the fact that IBS accounts for 20%-50% of referrals to gastrointestinal clinics, the majority of IBS patients choose not to consult a doctor [2]. Using a precoded questionnaire, Thompson and Heaton discovered in 1980 that 14% of IBS patients had spastic colon and abdominal pain that was eased by defecation [3]. IBS is known to affect approximately 11.2% of the world's population. South America has the highest prevalence of IBS (21%), while Southeast Asia has the lowest prevalence (7%). Although the differences found are not statistically significant, the prevalence of IBS appears to decrease somewhat with increasing age. However, the rates of IBS are considerably lower in individuals aged 50 or older compared to those younger than 50. About 50% of patients' symptoms start before age 35, while 40% of patients are between the ages of 35 and 50. Women's chances of developing IBS are just slightly higher than men's [4]. In addition, the indirect and direct costs of IBS are estimated to be around $20 billion annually in the United States and up to €3,000 yearly per capita in European countries with universal healthcare coverage, being comparable to other chronic diseases with high prevalence such as diabetes, persistent asthma, or obstructive pulmonary disease [5].

Although IBS is not generally life-threatening, it can cause troublesome symptoms for those affected and has been associated with an increased incidence of depression and anxiety, thereby leading to severely reduced quality of life (QoL) [6]. IBS is characterized by a number of symptoms, including abdominal pain, altered stools, distention, bloating, straining, and urgency [7,8]. Generally, the clinical picture is that of altered stool form or frequency, which is usually seen along with abdominal pain or discomfort [9,10].

Drugs such as antispasmodics, antidepressants, opioids, H1 blockers, serotonin antagonists, and GABAergic agents have all been tried to treat IBS, but pharmacotherapies have not been shown to alter the long-term or natural history of the disorder and are therefore not a viable long-term solution to the problem [11].

Quite a few patients have observed food as a triggering factor for the development of IBS symptoms. Studies show that up to 84% of IBS patients report food-related symptoms [12]. One study even showed that almost half of the patients suffering from IBS suffer from worsening symptoms postprandially within 90 minutes of eating [13]. The form and nutrient content of ingested food can cause a number of symptoms through a variety of different mechanisms, such as bacterial fermentation that changes the gut flora, induction of specific osmotic load effects in the small bowel and colon, production of gas in the digestive tract, and alteration of immune responses [14,15]. Due to the drawbacks of drug therapy and the protracted nature of IBS, diet seems to be a key component of IBS management. IBS patients can benefit from a variety of dietary restrictions, including low reduced fermentable oligosaccharide, disaccharide, monosaccharide, and polyol (FODMAP) diet (LFD), gluten-free diet (GFD), starch- and sucrose-reduced diet (SSRD), and IgG-exclusion and fasting [16]. Lately, much importance is being given to diets with reduced FODMAPs. The aim of this article is to explore the relevance of diet in the pathophysiology and management of IBS, with a major focus on FODMAPs and how they affect those with IBS.

## Review

Low FODMAP diet

Evidence that lactose, fructose, and sorbitol, three poorly absorbed, short-chain carbohydrates, are responsible for the onset of IBS symptoms and that dietary restriction alleviates symptoms accumulated over the 1980s and 1990s [17]. However, over time, it was evident that these sugars were not the sole cause. On further research and examination of known literature about the biochemistry and physiology of other carbohydrates, it was found that galacto-oligosaccharides (GOS) and fructo-oligosaccharides (FOS) (fructans) are likewise short-chain carbohydrates that are only partially absorbed in the human gastrointestinal system. Potential offenders also included the partially absorbed sugar polyols sorbitol and mannitol, which are both naturally occurring in foods and are utilized as artificial sweeteners. The acronym FODMAPs is the consequence of grouping these short-chain, poorly absorbed carbohydrates by the length of their chains [18].

FODMAPs were first described in a paper published by Gibson and Shepherd in 2005 [19]. The first research trial that confirmed the role of an LFD in reducing gastrointestinal complaints was conducted in 2006. This study examined 62 people on a low-fructose/fructan diet who had IBS and fructose malabsorption in the past. On this food plan, 74% of patients overall reported symptom relief [20]. A follow-up, randomized, placebo-controlled rechallenge trial was conducted over a five-month period in 25 patients with IBS and fructose malabsorption, which confirmed the effectiveness of the diet [21]. The symptoms of all patients significantly worsened when they were exposed to fructose or fructans again, and they were further aggravated when fructose and fructans were consumed together. All patients improved on a low-fructose/fructan diet. There was little placebo effect [21].

Later, the mechanism by which FODMAPs exerted their effects was investigated using two different trials. The first was in 2010, where Barrett et al. used an ileostomy model to confirm that FODMAPs in meals are poorly absorbed in the small intestine. Increased water content in the output after FODMAP delivery to the stoma suggests an osmotic effect of the carbohydrates. This could very well be the physiological mechanism causing some people to get diarrhea [22]. In the second trial, IBS patients and healthy participants had their breath hydrogen levels measured while on low- and high-FODMAP diets. An LFD dramatically decreased the production of breath hydrogen in both healthy volunteers and IBS patients, which had a knock-on effect on the severity of gastrointestinal symptoms in the IBS population. This demonstrates the short-chain carbohydrates' fermentative properties and their contribution to the development of bloating, distension, stomach pain, and exaggerated flatulence [23].

These mechanistic findings are compatible with what is known currently about the pathophysiological processes of IBS. Visceral hypersensitivity causes the enteric nervous system to change its normal motility patterns in response to gut distension, which the brain may interpret as bloating, discomfort, or pain. The LFD lessens gas production and fermentation, which is likely to lessen the bloating brought on by food and thus lessen the severity of symptoms. Alterations in the gut microbiota's number, composition, function, and location are additional potential contributing factors. Small intestinal bacterial overgrowth (SIBO), which can cause fermentation of improperly absorbed carbohydrates in the small intestine's confined lumen and cause abdominal pain and discomfort, may occur in some IBS patients. They may have a higher concentration of methane-producing bacteria, which, when fermenting malabsorbed carbohydrates, produces methane gas, which has been linked to delayed transit and constipation [24,25].

Since these initial studies, more information on the chemical makeup of food has become accessible, allowing for a more refined FODMAP strategy. In addition to fructose, lactose, and fructans, a wider variety of FODMAPs, such as GOS, sorbitol, and mannitol, have been taken into account. The LFD as it stands today consists of these six carbohydrates, with published tables of food composition available for fruits, vegetables, breads, and cereals [26]. The summary of the highest FODMAP food sources from published food composition papers has been listed in Table 1.

**Table 1:** FODMAP carbohydrates and their richest food sources FODMAPs: fermentable oligosaccharides, disaccharides, monosaccharides, and polyols, GOS: galacto-oligosaccharides

FODMAPs	Richest food sources
Fructans	Wheat, rye, onions, garlic, artichokes
GOS	Legumes
Lactose	Milk
Fructose	Honey, apples, pears, watermelon, mango
Sorbitol	Apples, pear, sugar-free mints/gums
Mannitol	Mushrooms, cauliflower, sugar-free mints/gums

Not all patients will be affected by FODMAPs as symptom triggers. The only sugars likely to contribute are those which are malabsorbed. It is significant to note that GOS and fructans are almost always malabsorbed, and gut bacteria invariably ferment them as well [27]. Healthy individuals experience increased gas production and related flatulence as a result, whereas in individuals with IBS, the consequence can result in more severe symptoms due to changed gut flora, motility difficulties, and hypersensitivity [23]. As for the remaining FODMAP carbs, only those IBS patients who are malabsorptive to them will have symptoms. Sorbitol and mannitol are only partially absorbed. Most people can effectively absorb the little amounts contained naturally in foods, sugar-free goods, and medications [28]. In individuals with IBS, hydrogen breath testing after 10 g of sorbitol and mannitol revealed malabsorption in 57% and 20% of patients, respectively [29]. For all sugars, sorbitol, mannitol, fructose, and lactose malabsorption affects more than 18% of healthy individuals worldwide; the absence of changed gut microbiota and hypersensitivity explains why these individuals, despite malabsorption, don't have any symptoms [29,30].

An LFD is prescribed to individuals suffering from IBS in an attempt to remove the offending agents and prevent symptom formation. Foods that are permitted and prohibited in an LFD are listed in Table 2 [31].

**Table 2:** Allowed and forbidden foods

Food categories	Allowed foods	Forbidden foods
Cereals	Rice, porridge, oats, quinoa, tapioca, millet, amaranth, buckwheat, gluten-free bread and cereals, potato flour	Bread and bakery products, biscuits, croissants, pasta, wheat flour, Kamut, barley, rye, couscous, flour, muesli
Milk and derivatives	Lactose-free milk, rice milk, oat milk, soy milk and all vegetable drinks, yogurt (lactose-free, soy, Greek), hard cheeses, fruit sorbets	Cow milk, goat milk, yogurt with lactose, fresh cheeses, butter, ice cream, cream
Vegetables	Carrot, pumpkin, Chinese cabbage, celery, lettuce, spinach, potato, tomato, zucchini, eggplant, green bean, beets, red pepper, herbs, olives, bamboo shoots, fresh herbs	Asparagus, cauliflower, garlic, onion, shallot, mushroom, leek, chicory, fennel, artichoke, Brussels sprout, broccoli, radish, pepper, turnips
Legumes	Peas, soy products	Beans, chickpeas, lentils, soybeans
Fruits	Banana, blueberry, strawberry, raspberry, grape, melon, grapefruit, kiwi, orange, lemon, limes, pineapple, passion fruit	Apple, pear, watermelon, mango, apricot, avocado, cherry, peach, plum, persimmon, lychee, fruit juices
Dried fruits	Almonds, hazelnuts, walnuts, pine nuts	Pistachios, cashews
Sweeteners	White sugar, brown sugar, maple syrup	Agave, honey, fructose, xylitol, maltitol, mannitol, sorbitol

**Figure 1:**
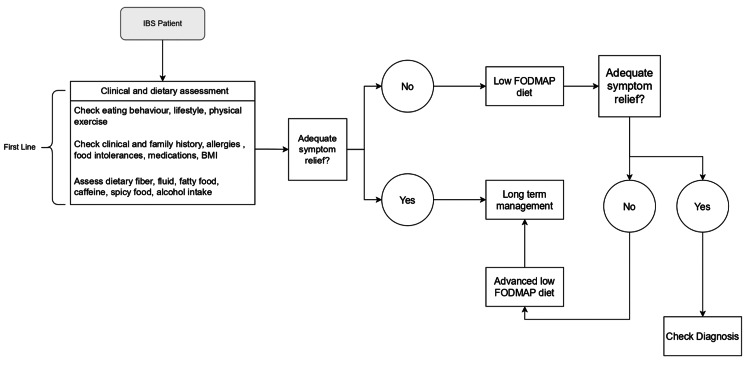
Low FODMAP dietary management algorithm in IBS IBS: irritable bowel syndrome, BMI: body mass index, FODMAPs: fermentable oligosaccharides, disaccharides, monosaccharides, and polyols, BMI: body mass index

An LFD consists of two phases. The first phase lasts for four to eight weeks, involves the complete elimination of all these molecules, and is followed by a second phase that involves the gradual reintroduction of one group of these carbs step by step. This enables the patient, who must be monitored by a qualified nutritionist, to recognize the types and quantities of meals to which they are hypersensitive and to devise suitable substitutions. Through this method, the doctor can individually modify the diet for each patient. It also guarantees that the diet is followed for the long term, creating an adapted low FODMAP diet (AdLFD) and reducing the likelihood of certain nutritional deficiencies [32]. Figure 1 outlines the FODMAP dietary management algorithm that is commonly used to create a diet plan that can be personalized for every patient [33].

It is always advisable to involve a qualified dietitian. This is due to the possibility that patient information about "trigger" meals that contain FODMAPs may not be accurate, even in a controlled gastroenterological setting, and therefore, the involvement of a qualified professional will help in providing more accurate information. Alternatively, mobile app-based tracking of food habits and reactions could also be done to identify the possible triggers when a professional cannot be consulted.

Despite the obvious advantages of the LFD, it is not without fault. The diet has recently been raising questions since it may have unintended consequences on the gut microbiota, which has been linked to the development of IBS [18]. Since IBS patients are known to have dysbiotic microflora, further alterations to the microbiota brought on by a FODMAP restriction diet may put the patient at risk for developing more pathological dysbiosis. The microbiota of IBS patients who underwent a four-week dietary intervention were compared to an IBS patient who followed a usual diet in a randomized controlled experiment. After the carbohydrate restriction, the authors showed a decrease in luminal Bifidobacteria concentration and percentage [34]. Recent randomized controlled trials also reported that an LFD can cause Bifidobacteria levels to decrease [35,36].

Therefore, evidence from the literature suggests that the LFD causes noticeable changes in the taxonomic composition of the gut microbiota. However, greater studies are required to determine whether these changes are harmful to health and whether they last for a long time. Therefore, in order to reduce the negative effects of altered gut flora, all patients are encouraged to try reintroducing FODMAPs at a level they can tolerate.

Gluten-free diet

Gluten is the common term for a family of proteins called prolamins (glutenin and gliadin), which are storage proteins in the starchy endosperm of many cereal grains such as wheat, barley, and rye. It is well known and understood in the setting of coeliac disease. The general public is increasingly restricting their gluten intake for various conditions, such as treating IBS, attention-deficit/hyperactivity disorder (ADHD), and chronic fatigue syndrome.

Although no evidence supports the use of a gluten-free diet for any condition other than celiac disease, alternative health practitioners frequently recommend it for IBS. A gluten-free diet has been reported to reduce symptoms in many IBS patients, but this was not previously researched. It is challenging to choose and research IBS individuals who benefit from a gluten-free diet because many have not had rigorous celiac disease testing before beginning the diet, which can confound the findings.

The first randomized controlled trial to demonstrate the occurrence of non-coeliac gluten intolerance was published by Biesiekierski et al. in 2011 [37]. Thirty-four IBS patients who had their symptoms under control on a gluten-free diet were given the option of eating gluten-containing bread and muffins or a placebo. In comparison to 40% of patients who received a placebo, 68% of patients experienced an aggravation of symptoms within one week of the gluten challenge [37]. This shows that IBS patients may be gluten intolerant, although no underlying mechanism was discovered. To confirm these results and understand the physiology of gluten intolerance in this patient group, more dietary experiments are necessary.

SSRD

The consumption of sugar has significantly increased during the previous few decades [38].

The enzymatic and absorptive systems are more highly upregulated at higher luminal sugar concentrations, which leads to increased glucose absorption [39]. As a result, increasing sugar consumption has a variety of effects on homeostasis, which in turn has an impact on health [40]. Reduced consumption of starch and sugar not only affects GI symptoms but also reduces weight and lowers the chance of developing metabolic disorders [41]. There must be a direct connection between the diet's effects on the intestine because cutting back on carbohydrates was associated with a decrease in GI symptoms [42].

SSRD has recently been demonstrated to significantly lessen GI symptoms in IBS patients, with a response rate of 74% [43]. Reducing the intake of carbs, disaccharides, starch, sucrose, and sugar was associated with a decrease in GI symptoms [42]. After the SSRD was introduced, lower levels of C-peptide, insulin, GIP, and leptin were seen, but the hormonal alterations were only linked with lower carbohydrate intake and weight, not with lower GI symptoms [44]. Diet had no impact on the amount of inflammatory substances in the blood.

The mechanisms underlying the effect of SSRD in IBS are unknown, but three different hypotheses are tenable explanations for GI symptoms following the consumption of starch and sucrose [45]. First, IBS has been linked to functional variations of the enzyme sucrase-isomaltase (SI). Thus, a subgroup of IBS patients may experience partial SI deficit with impaired starch and sucrose digestion. Second, after consuming large amounts of sucrose, physiological fructose malabsorption may occur because fructose is less efficiently absorbed than glucose. Third, diets low in starch and sugar cause weight loss and decreased levels of C-peptide, insulin, gastric inhibitory peptide, and leptin. The lessened symptoms observed after the diet may be caused by these metabolic changes, which may lessen the excitability of the hypersensitive nervous system frequently present in IBS.

The SSRD has the advantage that GI symptoms are improved quickly compared to other diets and that there are fewer foods to cut out from one's diet, making it easier to follow.

An LFD advises limiting lactose intake and eliminating wheat and rye from cereals, which is one of the biggest distinctions between it and the SSRD.

Although some level of improvement has been seen following the SSRD, more research is required to understand the pathophysiology of symptom development following starch and sucrose intake, as well as the mechanisms underlying symptom alleviation following reduced intake. Future research should examine the role of dietary variables, such as sugar, in the development of low-grade inflammation and increased gut permeability in the emergence of IBS and FGID.

Prebiotics, probiotics, and synbiotics

Probiotics have been defined in 2001 by the Food and Agriculture Organization (FAO) and World Health Organization (WHO) as "live microorganisms, which when administered in adequate amounts, confer a health benefit on the host" [46]. They exhibit a variety of favorable physiological effects in the gut, enabling their use as an IBS treatment.

The mechanisms of action of probiotics at the gut level include modulating the gut microflora through competition and inhibition of pathogen adhesion to the gut epithelia by the production of bacteriocins, short-chain fatty acids (SCFAs), and biosurfactants; decreasing low-grade mucosal immune activation, increasing the mucus layer, and producing proteins of tight junctions, thereby improving the gut mucosa's ability to function as a barrier; suppressing pro-inflammatory cytokines, which has an anti-inflammatory effect; stimulating secretory IgA production; and improving gut-brain communication [47,48].

The importance of the gut microbiota in the pathophysiology of IBS has been highlighted by recent studies and clinical evidence, which has prompted the use of novel therapies such as prebiotics, probiotics, synbiotics, and fecal microbiota transfer (FMT) that aim to modify the gut microbiota toward a beneficial composition for IBS patients [49-51].

In 2016, the International Scientific Association for Probiotics and Prebiotics (ISAPP) defined prebiotics as a "substrate that is selectively utilized by host microorganisms conferring health benefit" [52]. The term "prebiotic" refers to non-viable dietary components such as fructan (e.g., inulin), indigestible polysaccharides, GOS, oligosaccharides, or fructo-oligosaccharides (FOS), which preferentially stimulate the growth of a small number of health-promoting bacteria in the colon and exert health benefits that can include positive effects on the GI tract, brain function, cardiovascular health, and bone strength.

Despite the fact that an LFD frequently reduces IBS symptoms, it does not offer long-term relief, and symptoms typically return once the diet is stopped. Additionally, eliminating all fermentable oligosaccharides from the diet may have a negative impact on long-term health by upsetting the balance of the gut's microbial ecosystem. It has been demonstrated that continuous prebiotic (GOS) supplementation can cause less gas production and more tolerability in vivo [53], and in patients with functional gut disorders, prebiotic supplementation may be superior to an LFD [54].

In May 2019, a panel of experts at the International Scientific Association for Probiotics and Prebiotics (ISAPP) defined synbiotics as "a mixture comprising live microorganisms and substrate(s) selectively utilized by host microorganisms that confers a health benefit on the host" [55]. In accordance with ISAPP, synbiotics are being employed as nutritional and medicinal supplements as the synergistic effects of synbiotics comprise the selectivity of prebiotics toward probiotic metabolism, which ensures their survival and development in the gut [55,56]. The rationale for the development of synbiotics is that probiotics are sensitive to changes in growing conditions such as pH, oxygen, and temperature and cannot thrive in the human digestive system without the presence of prebiotics.

Two general methods by which synbiotics might enhance the effects of their ingredients are outlined by Kolida and Gibson [57] and Swanson et al. [55]. The first method is that the components of complementary synbiotics are supplements that are selected separately, with each being in charge of their own specific effect. In this scenario, the prebiotic may not be preferentially digested by the probiotic strain and may instead be fermented by the host's microbiota. The synergistic synbiotics, on the other hand, are an example of the second method outlined, where the prebiotics are chosen with care to serve as a substrate for the probiotic strains and to promote their growth [55].

A growing amount of research shows that probiotics and prebiotics have a modulatory effect on the human intestinal microbiota, changing it to have a healthier composition for the host.

Different responses to the same probiotic administration were observed in IBS patients with different clinical profiles, suggesting that a number of variables, including age, gender, diet, bowel habits, the composition of the microbiota, and the presence of psychological comorbidities, may influence and serve as useful predictors of the outcomes of probiotic interventions [4,58,59].

The American College of Gastroenterology (ACG), however, noted in the most recent treatment monograph [60] that there is very weak support for prebiotics and synbiotics in IBS due to the low quality of evidence supporting these treatments. The failure to disclose the technique employed to hide treatment allocation and the high variation between studies were the primary causes of the uncertain risk of bias in double-blinded clinical trials. A study conducted by Ford et al. in 2018 also concluded that there was little evidence for the use of prebiotics or synbiotics in IBS [61]. Because the synergistic and supportive mechanisms involved in the enhancement of host health are not fully understood, synbiotics present a significant research challenge.

However, despite the drawbacks, probiotic and prebiotic research should still be explored because their administration in combination as synbiotics has opened up a new area of investigation in the realm of complementary therapy for IBS.

## Conclusions

IBS continues to be a mysterious source of severe distress, illness, and impairment. In the near future, diagnosing IBS will depend on identifying its distinctive symptoms and ruling out organic disease mimics. A better knowledge of its etiology will also facilitate the development of new non-pharmacological and pharmaceutical treatments for IBS. GFD, SSRD, and diet supplements such as prebiotics and probiotics have shown considerable uses to tackle the disease, but none of them have shown results as consistently and neither have they been as thoroughly studied as the LFD, and the LFD does appear to be a more successful symptom-reduction strategy when compared to other dietary and non-dietary therapies. However, why some individuals respond well to some therapies while others do not is still a mystery. The complexity of IBS makes it difficult to develop a comprehensive, effective course of treatment. The lack of an effective IBS treatment and the wide range of therapies that have been tried clearly indicate that the pathophysiology of the illness has not yet been fully explored. Since the disease affects a large chunk of the general population, for the time being, it is critical for doctors to appreciate the role of dietary, lifestyle, and behavioral changes for IBS, whether these changes are combined with or independent of medicinal treatments, and future studies regarding this subject must be promoted in order to understand the disease process and find a proven solution to tackle the problem at hand.
